# Effectiveness of a Stigma Awareness Intervention on Reemployment of People with Mental Health Issues/Mental Illness: A Cluster Randomised Controlled Trial

**DOI:** 10.1007/s10926-023-10129-z

**Published:** 2023-07-13

**Authors:** K. M. E. Janssens, M. C. W. Joosen, C. Henderson, M. Bakker, W. den Hollander, J. van Weeghel, E. P. M. Brouwers

**Affiliations:** 1 Scientific Center for Care and Wellbeing, Tilburg School of Social and Behavioral Sciences, Tilburg University, Tilburg, The Netherlands; 2 Health Services and Population Research Department, King’s College London Institute of Psychiatry, Psychology and Neuroscience, London, UK; 3 Department of Methodology and Statistics, Tilburg School of Social and Behavioral Sciences, Tilburg University, Tilburg, The Netherlands; 4 Department of Epidemiology, Data & Monitoring, Trimbos Institute, Utrecht, The Netherlands; 5 Phrenos Center of Expertise, Utrecht, The Netherlands

**Keywords:** Mental health issues, Mental illness, Stigma, Discrimination, Employment rates

## Abstract

**Purpose:**

A barrier for reemployment of people with mental health issues/mental illness (MHI) is workplace stigma and discrimination. In this RCT the effectiveness of a stigma-awareness intervention addressing finding work, retaining work and decisional stress were evaluated.

**Methods:**

A cluster RCT was conducted in 8 Dutch municipal practices. Randomisation took place at practice level. Participants were unemployed people with MHI, receiving social benefits. The intervention consisted of a decision aid for workplace disclosure for participants and a 2 × 3 h stigma-awareness training for their employment specialists. Primary outcomes were measured at baseline, 3-, 6- and 12-months. Multilevel analyses, containing random intercepts of participants nested in organizations, were conducted to analyse the effects of the intervention.

**Results:**

Participants (N = 153) were randomized to an experimental (n = 76) or control group (n = 77). At six months, significantly more participants of the experimental group (51%) had found work compared to the control group (26%). At twelve months, significantly more participants of the experimental group (49%) had retained work compared to the control group (23%). Intention-to-treat analyses showed that randomization to the experimental group was associated with finding (OR(95%CI) = 7.78(1.33–45.53), p = 0.02) and retaining (OR(95%CI) = 12.15(2.81–52.63), p < 0.01) work more often at twelve months. Analyses showed that the experimental and control group did not differ in decisional stress.

**Conclusions:**

Our stigma awareness intervention was effective for finding and retaining work. As the percentage of people who found and retained work almost doubled, this suggests that on a societal level, a vast number of unemployed people could be reemployed with a relatively simple intervention.

**Trial Registration:**

The study was retrospectively registered at the Dutch Trial Register (TRN: NL7798, date: 04-06-2019).

**Supplementary Information:**

The online version contains supplementary material available at 10.1007/s10926-023-10129-z.

## Introduction

People with mental health issues/mental illness (MHI) are three to seven times more likely to be unemployed than people without MHI [1]. This is problematic, because being employed contributes to health and recovery [2]. Also, unemployment has been associated with poorer (mental) health [3–5], poverty [4] and higher risk of suicide [6]. Employment has many benefits for mental health, such as time structure, purpose and having a daily activity, and financial problems because of insufficient income [3, 7]. Contrarily, (re-)employment, provided under favourable conditions, improves health, as well as self-esteem, mastery and happiness [8], and enhances recovery of MHI on several dimensions, such as functional, existential and social recovery [9].

A major barrier for people with MHI is workplace stigma and discrimination [10, 11]. Both (negative) attitudes and behaviours of employers, as well as anticipated stigma and self-stigma in people with MHI are obstacles in finding and keeping employment [12]. For instance, a recent representative study found that 64% of Dutch managers were reluctant to hire a job applicant with MHI, and 30% were even reluctant to hire an applicant who has recovered from MHI [13]. Moreover, having experienced discrimination because of MHI has shown to negatively influence job searching activities [14]. Recent studies have highlighted the importance of disclosure decisions for re-employment success in people with MHI [10, 15–17].

The decision whether or not to disclose a MHI in the work context is a very personal and complex one. Disclosure can have beneficial outcomes, e.g. co-worker support and work adjustments, that may help retain employment during difficult times [17]. In contrast, disclosure can also have adverse outcomes such as stigma and discrimination, which may damage careers and lead to job loss. Non-disclosure can also have positive effects (the avoidance of stigma and discrimination) as well as negative effects (not receiving support and work adjustments that are needed) [12, 17, 18]. Several recent studies have suggested that the decision regarding disclosure can impact the reemployment success of people with MHI [15]. For instance, a pilot randomised controlled trial (RCT) showed that people who used the CORAL (Conceal or Reveal) decision aid [19] were more often working full time after 3 months than people who did not use the decision aid and experienced less decision-making stress [16]. Although there are strong indications that stigma and discrimination negatively impact employment opportunities [10, 13] and disclosure decision aids seem promising [16, 20], longitudinal research on the long-term employment outcomes of disclosure decisions for people with MHI is lacking [10].

Therefore, in this RCT the effectiveness of a stigma awareness intervention on reemployment and decisional stress was evaluated in unemployed people with MHI. This intervention aimed to increase awareness about stigma and the importance of a deliberate disclosure process in both unemployed people with MHI and the employment specialists who support them in their vocational rehabilitation trajectory. The primary aim of this study is to evaluate at 3, 6 and 12 months after baseline whether this stigma awareness intervention led to (1) finding paid employment more often (yes/no); (2) retaining paid employment more often (yes/no); and (3) less decisional conflict about disclosing MHI, compared to usual vocational rehabilitation in municipal practice. An additional aim was to gain insight into the (long-term) effects of the intervention compared to usual vocational rehabilitation on secondary outcomes, such as mental health and stigma.

## Method

### Study Design

The DECIDES (DECIsions on Disclosure in the Employment Setting) study is a longitudinal, two-armed, clustered RCT among unemployed people with MHI who receive social benefits and reintegration support from Dutch municipalities. More details of the study design and measurements of the RCT have been reported in a study protocol (https://doi.org/10.1186/s13063-020-04376-1) [21]. The Ethics Review Board of Tilburg University evaluated and approved the study design, protocol, information letter, informed consent form and questionnaires (EC-2018.06t). The study was designed and analysed following the ‘CONSORT 2010 statement: extension to cluster randomized controlled trials’ [22] and registered at the Dutch Trial Register under trial registration number NL7798.

### Setting

The current study took place in the southern part of the Netherlands (province of Noord-Brabant). At the time of conducting the study, unemployment rates were around 3.2% for Noord-Brabant compared to around 3.5% of the working population for the Netherlands [23]. People who have insufficient income or capital and have no rights on other provisions or benefits (such as unemployment benefits) are entitled to social benefits. In the Netherlands, these social benefits are paid out by municipalities but in order to receive those several obligations must be met, such as putting in enough effort to try to enter the job market. In case income is received, e.g. by finding paid employment, this will be deducted from the social benefits. Municipalities organise their own vocational rehabilitation services, consisting of various facilities, such as support from employment specialists, education facilities and training.

### Randomisation

**Fig. 1:**
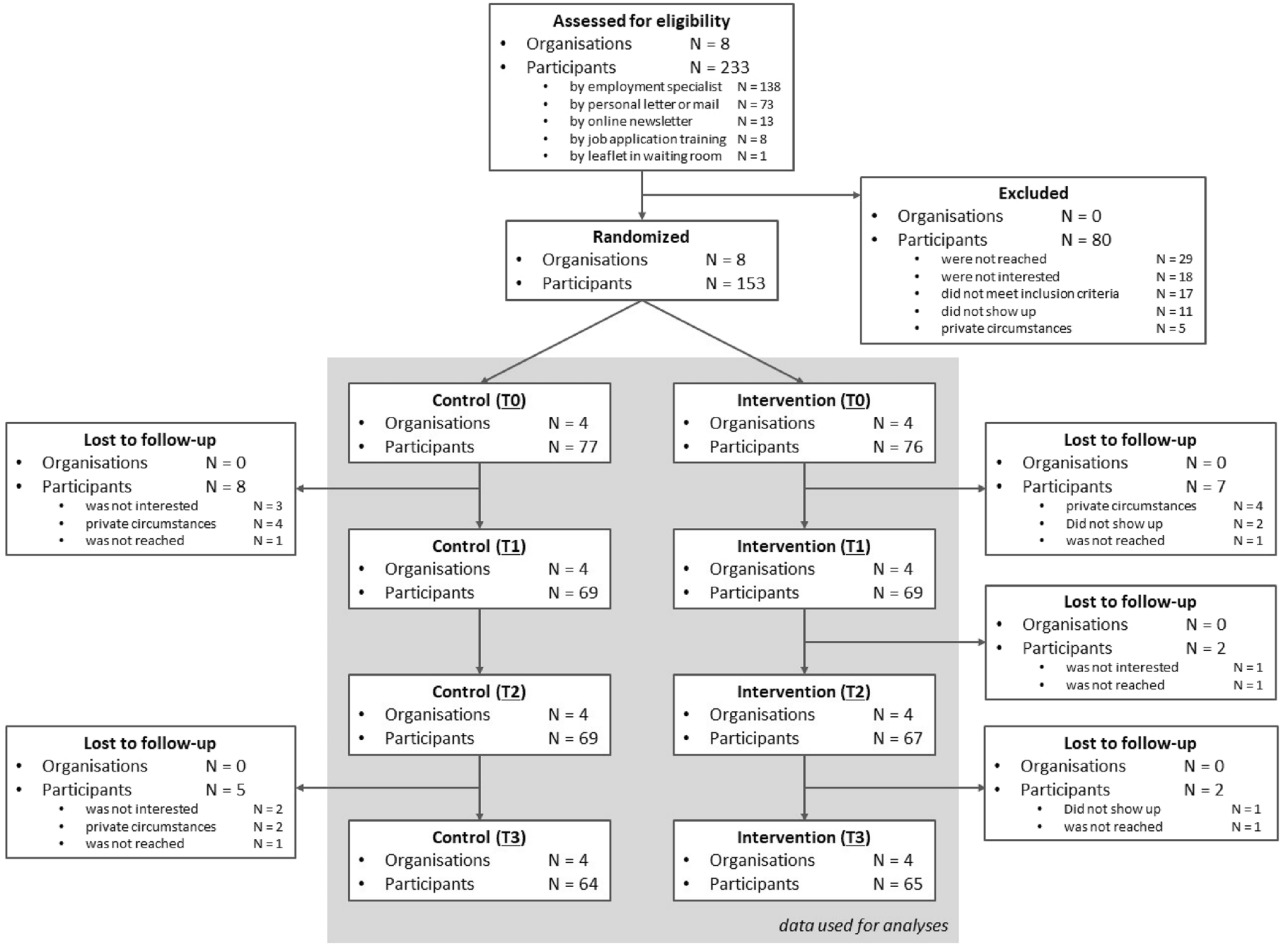
Overview of the randomisation process

This study consists of the conditions (a) vocational rehabilitation as usual (i.e. control group) and (b) vocational rehabilitation as usual combined with the intervention (i.e. experimental group). Cluster randomisation on organisation level was chosen to avoid contamination between the experimental and control group, as employment specialists in the organisations work intensively together in teams. Organisations were municipalities and organisations commissioned by municipalities in the southern part of the Netherlands. Randomisation into the control or experimental group was conducted by a researcher who was not involved in the research project, by computer allocation using SPSS-software. Within the participating organisations, employment specialists (N = 72) were recruited between November 2017 and March 2018. Due to the cluster design of the study and the nature of the intervention, neither the employment specialists nor the researchers could be masked to the allocation to the conditions, However, employment specialists and participants of the control group were not informed about the content of the intervention. Figure 1 gives an overview of the randomisation process.

### Participants

Participants were recruited by N = 72 employment specialists within the eight participating organisations during personal contacts with clients, via newsletters, and personal letters to potential participants. Inclusion criteria were (1) being unemployed, i.e. an income below minimum income and receiving social benefits, (2) having sought any treatment (currently or in the past) for MHI, or addiction, from a health professional (e.g. general practitioner, psychologist) and (3) adequate command of the Dutch language, as the intervention and questionnaires were in Dutch. Employment specialists were asked to provide information about the research to people who met the inclusion criteria. People who were willing to receive more information about the research and gave permission for their contact details to be shared with the researchers were contacted. Here, inclusion criteria were also checked. Participation in the study was voluntary. All participants signed an informed consent form prior to participation.

### Procedures

Measurements took place at baseline (T0), after 3 months (T1), 6 months (T2) and 12 months (T3). Participants could start at any moment during the recruitment period and were followed from then on until 12 months later. As participants included people with literacy and concentration difficulties who could drop out of a longitudinal study, extra efforts were made to recruit and retain them, e.g. by gathering the data during face-to-face appointments. Participants received a financial remuneration of 10 euros (8.5GBP) after filling out each questionnaire.

The intervention consisted of two parts: (1) a printed version of the CORAL.NL for participants, together with two infographics providing a brief and simplified version of the CORAL.NL tool, designed for participants with literacy or concentration problems and (2) a workplace stigma-awareness training for employment specialists.

The workplace stigma-awareness training for employment specialists was developed for the purpose of this study, using literature on effective elements of stigma interventions [24–28] and input from a focus group study with five stakeholder groups: i.e. people with lived experience, human resources professionals, employers, employment specialists and mental health advocates [17]. Findings of this study were implemented in both the workplace stigma awareness training for employment specialists as well ass the Dutch version of the CORAL decision aid.

The stigma awareness training for employment specialists had several aims: () creating awareness of stigma and discrimination in the work environment and creating insight into the effects of employment specialists’ own attitudes, personal prejudices and actions; (2) increasing understanding of how the disclosure dilemma can be experienced by people with MHI and how it affects them, and (3) learning to work with the CORAL.NL decision aid and infographics, including how they can be implemented in daily practice. The full training consisted of three meetings within 6 months. Each meeting has a duration of 2 h and was provided in groups of 4–12 employment specialists under guidance of 2–3 trainers. One of the effective elements is personal contact with someone with lived experience in various forms, such as by attending a presentation or interview with someone with lived experience [24]. Therefore, during the first training session, an (semi-structured) interview was organized with a mental health advocate with lived experience. This interview was held by one of the trainers, and the employment specialists also had the opportunity to ask questions during or after the interview. During the second training session, a film with personal stories of five workers with lived experiences who had been stigmatized or discriminated in the work environment was displayed as evidence indicates that also filmed material can be effective in destigmatizing interventions [29]. Finally, during the third (last) training session, role plays were organized between the employment specialists and a mental health advocate with lived experience to practice the conversation about the disclosure dilemma.

The CORAL.NL decision aid is based on the Conceal Or ReveAL (CORAL) decision aid, developed and tested in the UK [16]. Subsequently, CORAL was translated and developed further into the CORAL.NL for the Dutch practice by conducting a focus group study [17]. Adjustments were related to Dutch legislation (i.e. in the Netherlands, an employer is not allowed to ask questions about the nature and cause of an employee's illness), and by including more information about who to disclose to, timing, preparation, message content and communication style as these were important topics found in a previous focus group study [17]. In addition, internet links were added to web pages with more information about the Dutch (legal) system. Similar to the English version, the CORAL.NL decision aid consists of a 14-page booklet containing four parts. Part 1 deals with choices about disclosure, the pros and cons of disclosure, and personal disclosure needs and values. Part 2 is about one’s personal situation and deals with questions about to whom and when to disclose. Parts 3 and 4 summarize previous sections to make a plan about whether to disclose or not, and if so, to whom and when and what to disclose. As participants included people with concentration and literacy problems, for whom a 14-page booklet may not be suitable, two very brief infographics were developed, summarizing pros, cons and tips regarding disclosure during the job application process and during employment, respectively. Participants of the experimental group received the CORAL.NL decision aid and infographic from the researcher after filling out the baseline questionnaire (T0) (see online appendix 1–4).

### Measurements

Primary outcomes were: (1) finding paid employment (yes/no), defined as a minimum of one hour a week for a minimum of one month; (2) retaining paid employment (yes/no), i.e. at least 12 h a week, for a minimum of three consecutive months; and (3) decisional conflict, measured with the 17-item Decisional Conflict Scale [30], and the one-item Stage of Decision Making Scale [31], which measures the individuals’ readiness to engage in decision making. Primary outcomes were measured at each data collection point.

In addition, secondary variables were: (1) self-reported current mental health, measured with the Patient Health Questionnaire [32, 33], a screening tool with good diagnostic validity for mental health disorders of somatoform, depression, anxiety, alcohol, and eating disorders; (2) positive wellbeing, which is measured with the 14-item Warwick-Edinburgh Mental Wellbeing Scale (WEM-WBS), a scale with good content validity and test–retest reliability [34]; (3) internalized stigma, using the 10-item Internalized Stigma of Mental Illness Scale (ISMI-10) which has good internal consistency [35, 36]; (4) experienced discrimination, measured with two specific items from the Discrimination and Stigma Scale (DISC-12) [37] about finding and keeping a job; (5) work-related factors, i.e. active in searching and applying for jobs and five statements on a 5-point Likert scale about personal fears about reemployment; and (6) the quality of support from employment specialists, using three items of the Patient Satisfaction with Occupational Health Professionals scale [38]. Secondary variables were measured at each data collection point. In addition, personal characteristics about age, sex, nationality, marital status, level of education and history of mental and physical ill-health were assessed at baseline.

### Statistical Analyses

Analyses were carried out using R, version 4.0.2 (R Program for Statistical Computing) and SPSS, version 27.0 (IBM Corporation). The effect size for the current clustered power calculation was based on data from a recent international study on individual placement and support [39] with a similar primary outcome measure, i.e. obtaining employment. In that study, the average percentage employment was 50% in the experimental group and 20% in the control group. Here, assuming an effect size of Cohen’s *d* = 0.67, a 2-sided α = 0.05 and an intraclass correlation coefficient (ICC) = 0.2 a repeated measurements analysis would require a minimum of 76 participants per arm to detect significant differences between groups with power β = 80% [40]. These results were verified by simulation using the R package *simr* [41], specifically simulating a binary outcome variable yielded an average power (95% CI) = 79% (70%-87%) after 1000 simulations.

All statistical analyses were performed according the intention-to-treat population by means of repeated-measures mixed models using the R package *lme4* [42] for continuous and binary outcomes (with logit link), and the R package *ordinal* [43] for ordinal outcomes, in which random intercepts were included denoting organisation. Effect sizes for continuous, binary and ordinal outcomes are reported as Cohen’s D, Odds Ratio and Odds Ratio respectively, with 95% confidence intervals and p-values. For the primary and secondary outcome analyses an additional random intercept was included denoting participant, to account for repeatedly measuring the same individuals each data collection point (*i.e.* the longitudinal nature of the study). The primary and secondary outcomes were analysed according to the recommendation of Twisk *and colleagues* [44], i.e. without the treatment variable, but with data collection point and interaction terms between treatment and data collection point as fixed effects in the models. We report on them at each data collection point separately using dummy variables, as well as in interaction with data collection point coded numerically.

## Results

Participants were recruited between April 10, 2018 and July 8, 2019. Of the 233 people screened, N = 153 participants met the inclusion criteria and were willing to participate (see Fig. 1). Sociodemographic characteristics and health characteristics were well balanced between the control and experimental group (see Table 1). Participants who dropped out during the study did not differ significantly from participants who completed all measures on the sociodemographic and health characteristics displayed in Table 1 (data not shown).

**Table 1:** Characteristics sample

	Control group (N = 77)			Experimental group (N = 76)		
	N (%)	M (95% CI)	M (SD)	N (%)	M (95% CI)	M (SD)
Age		40.01 (37.21–42.8)	40.01 (12.51)		37.4 (− 12.5–36.22)	37.4 (11.86)
Gender: female	37 (48.1)			44 (57.9)		
Nationality						
Dutch	71 (92.2)			73 (96.1)		
Other than Dutch	6 (7.8)			3 (3.9)		
Marital status						
No relationship (single, divorced or widowed)	65 (84.4)			62 (81.6)		
Relationship (married, relationship living apart or co-habitation)	12 (15.6)			14 (18.4)		
Educational status						
Lower educated or no education	31 (40.3)			39 (51.3)		
Medium educated	28 (36.4)			24 (31.6)		
Higher educated	18 (23.4)			13 (17.1)		
Self-reported diagnosis*						
Anxiety	13 (16.9)			6 (7.9)		
Attention deficit (hyperactivity) disorder	12 (15.6)			11 (14.5)		
Autism spectrum disorder (including asperger and PDD-NOS)	8 (10.4)			14 (18.4)		
Bipolar disorder	1 (1.3)			2 (2.6)		
Burnout, stress, overload	9 (11.7)			12 (15.8)		
Depression	23 (29.9)			20 (26.3)		
Personality disorder	14 (18.2)			11 (14.5)		
Psychotic disorder	2 (2.6)			3 (3.9)		
PTSD	11 (14.3)			12 (15.8)		
Schizophrenia	1 (1.3)			0 (0)		
Other	8 (10.4)			7 (9.2)		
Don’t know	7 (9.1)			7 (9.2)		
No diagnosis	8 (10.4)			11 (14.5)		
Percentage ever had chronic diseases (such as heart complaints, epilepsy)	33 (44)			38 (50)		
Percentage ever admitted to psychiatric hospital	15 (19.5)			11 (14.5)		
Percentage length of time out of employment > 12 months	49 (64.5)			48 (64.9)		

*Percentage is above 100% because of comorbidity

PDD-NOS Pervasive developmental disorder-not otherwise

First, regarding finding paid employment, at three months after baseline (T1) N = 23 (33.3%) participants of the experimental group had found paid employment, compared to N = 18 (26.1%) participants of the control group. This difference was not significant (OR (95% CI) = 2.21 (0.43–11.38), p = 0.34). After six months (T2), significantly more participants of the experimental group (N = 34, 50.7%) had found paid employment, compared to participants of the control group (N = 18, 26.1%; OR (95% CI) = 14.42 (2.47–84.07), p < 0.01). Similarly, after twelve months (T3), significantly more participants of the experimental group (N = 35, 53.8%) found paid employment than participants of the control group (N = 22, 34.4%; OR (95% CI) = 7.78 (1.33–45.53), p = 0.02; see Table 2). In parallel, across all data collection points collectively, significantly more participants of the experimental group found paid employment than participants of the control group (interaction OR (95% CI) = 2.16 (1.27–3.66); p = 0.004).

**Table 2:** Primary outcomes

	Control group		Experimental group		Cohen’s D (95% CI)	OR (95% CI)	P-value
	M (SD)	N (%)	M (SD)	N (%)			
Finding paid work							
T0		7 (9.1)		10 (13.2)			
T1		18 (26.1)		23 (33.3)		2.21 (0.43–11.38)	0.34
T2		18 (26.1)		34 (50.7)		14.42 (2.47–84.07)	** < 0.01**
T3		22 (34.4)		35 (53.8)		7.78 (1.33–45.53)	**0.02**
						Interaction OR (95% CI) = 2.16 (1.27–3.66)	** < 0.01**
Retaining paid work (12 h/w, 3 m)							
T0		0 (0)		0 (0)			
T1		4 (5.8)		9 (13)		4.92 (0.63–38.31)	0.13
T2		15 (21.7)		16 (23.9)		1.59 (0.34–7.43)	0.55
T3		15 (23.4)		32 (49.2)		12.15 (2.81–52.63)	** < 0.01**
						Interaction OR (95% CI) = 1.92 (1.18–3.12)	**0.01**
Decisional conflict							
T0	33.12 (17.28)		38.77 (20.32)				
T1	32.2 (16.03)		35.1 (21.14)		− 0.05 (− 0.32–0.24)		0.70
T2	31.84 (18.28)		33.06 (17.61)		− 0.13 (− 0.41–0.16)		0.35
T3	31.74 (15.76)		32.91 (19.1)		− 0.15 (− 0.42–0.15)		0.30
					Interaction Cohen’s D (95% CI) = − 0.05 (− 0.14–0.04)		0.24

P-values < 0.05 are in bold

Stage of decision making can be found in online appendix 2

Second, concerning retaining paid employment, at three months (T1), N = 9 (13.0%) participants of the experimental group had retained paid employment, compared to N = 4 (5.8%) participants of the control group. This was a non-significant difference (OR (95% CI) = 4.92 (0.63–38.31), p = 0.13). After six months (T2), N = 16 (23.9%) participants of the experimental group had retained paid employment, compared to N = 15 (21.7%) participants of the control group. Also this difference was not significant (OR (95% CI) = 1.59 (0.34–7.43), p = 0.55). However, After twelve months (T3), significantly more participants of the experimental group (N = 32, 49.2%) had retained paid employment compared to participants of the control group (N = 15, 23.4%; OR (95% CI) = 12.15 (2.81–52.63), p < 0.01; see Table 2). Alongside these data collection point specific comparisons, we observed an overall significant increase of retained paid work among participants in the intervention group when compared to the control group across all data collection points (interaction OR (95% CI) = 1.92 (1.18–3.12); p = 0.008).

At all measurements, no significant differences were found between control and experimental group on decisional conflict, including stage of decision making regarding disclosure (see Table 2 and online appendix 5), neither when analysing data collection points separately nor when testing for an overall intervention effect.

For the second aim of this study, the effects of the intervention on secondary variables were studied. No significant differences were found between control and experimental group on secondary outcomes in follow up measurements, except for somatoform disorder, number of positive indications for mental health problems, internalized stigma and quality of support. Significantly more participants of the experimental group had an indication for somatoform disorder than participants of the control group at all measurements. At six months, participants of the experimental group had significantly lower internalized stigma than the control group. In addition, at six months, participants of the experimental group were significantly more positive about the support, professionalism and overall support of their employment specialists than participants of the control group. At 12 months, participants of the experimental group had significantly less indications for mental health problems than the control group (see Table 3).

**Table 3:** Secondary outcomes

	Control group								Experimental group							
	T0		T1		T2		T3		T0		T1		T2		T3	
	M (SD)	N (%)	M (SD)	N (%)	M (SD)	N (%)	M (SD)	N (%)	M (SD)	N (%)	M (SD)	N (%)	M (SD)	N (%)	M (SD)	N (%)
*Secondary outcomes*																
Mental health problems (PHQ)																
Somatoform disorder		0 (0.0)		17 (24.6)		14 (20.3)		17 (26.6)		21 (27.6)		21 (30.4)		20 (29.9)		19 (29.2)
Depressive disorder		15 (19.5)		27 (39.1)		21 (30.4)		24 (37.5)		23 (30.3)		27 (39.1)		22 (32.8)		17 (26.2)
Anxiety disorder		23 (30.3)		16 (23.2)		19 (27.5)		12 (19.0)		17 (22.4)		15 (22.1)		20 (29.9)		13 (20.0)
Eating disorder		0 (0.0)		5 (8.1)		1 (1.6)		4 (7.1)		3 (4.1)		2 (3.1)		2 (3.3)		5 (8.1)
Alcohol abuse		13 (16.9)		6 (8.7)		6 (8.7)		8 (12.5)		5 (6.6)		6 (8.7)		4 (6.0)		5 (7.8)
Total number of positive indications	0.66 (0.82)		1.03 (1.04)		0.88 (1.06)		1.02 (1.11)		0.91 (1.05)		1.03 (1.12)		1.01 (1.01)		0.91 (1.11)	
Positive wellbeing (WEM-WBS)	47.19 (9.39)		48.2 (8.66)		47.9 (10)		47.08 (8.07)		47.3 (9.84)		47.7 (10.02)		47.58 (10.23)		47.8 (11.07)	
Internalized stigma (ISMI)	2.10 (0.44)		2.03 (0.42)		2.06 (0.44)		2.05 (0.42)		1.94 (0.45)		2.01 (0.45)		1.94 (0.45)		1.98 (0.45)	
Experienced discrimination finding work (DISC)																
Did experience discrimination		26 (33.8)		21 (30.4)		21 (30.4)		18 (28.1)		14 (18.4)		22 (31.9)		18 (27.3)		20 (31.2)
Did not experience discrimination		22 (28.6)		20 (29)		21 (30.4)		18 (28.1)		23 (30.3)		20 (29)		20 (30.3)		17 (26.6)
N/a (did not searched for work yet)		29 (37.7)		28 (40.6)		27 (39.1)		28 (43.8)		39 (51.3)		27 (39.1)		28 (42.4)		27 (42.2)
Experienced discrimination keeping work (DISC)																
Did experience discrimination		33 (42.9)		25 (36.2)		27 (39.1)		23 (35.9)		27 (35.5)		34 (49.3)		26 (39.4)		25 (39.1)
Did not experience discrimination		19 (24.7)		22 (31.9)		16 (23.2)		15 (23.4)		17 (22.4)		13 (18.8)		16 (24.2)		17 (26.6)
Keeping work: n/a (did not had a job yet)		25 (32.5)		22 (31.9)		26 (37.7)		26 (40.6)		32 (42.1)		22 (31.9)		24 (36.4)		22 (34.4)
Timing of disclosure																
Preferably disclosing MHP during application process		27 (35.1)		31 (44.9)		28 (40.6)		28 (43.8)		27 (36.0)		24 (35.3)		30 (44.8)		25 (39.1)
Preferably disclosing MHP at work (after application process)		10 (13.0)		9 (13.0)		8 (11.6)		9 (14.1)		8 (10.7)		12 (17.6)		12 (17.9)		12 (18.8)
Preferably not disclosing MHP at work		26 (33.8)		19 (27.5)		20 (29)		17 (26.6)		21 (28)		18 (26.5)		17 (25.4)		16 (25.0)
Don't know		14 (18.2)		10 (14.5)		13 (18.8)		10 (15.6)		19 (25.3)		14 (20.6)		8 (11.9)		11 (17.2)
Searched or applied for a job in the past four weeks		41 (53.2)		32 (46.4)		33 (47.8)		22 (34.4)		51 (67.1)		32 (46.4)		31 (46.3)		21 (32.3)
Received guidance from your employment specialist in the past 3 months (6 m for T3)		49 (79.0)		46 (74.2)		37 (59.7)		30 (48.4)		55 (85.9)		41 (64.1)		39 (60.9)		25 (39.1)
If yes:																
What grade would you give the guidance?	6.85 (2.28)		6.47 (2.76)		6.38 (2.51)		6.68 (2.40)		7.33 (2.33)		7.16 (2.21)		7.12 (2.44)		6.92 (2.78)	
The employment specialist treated me in a pleasant manner (based on PSO-HP)	4.38 (1.16)		4.27 (1.18)		4.00 (1.36)		3.97 (1.45)		4.53 (0.91)		4.58 (0.92)		4.55 (0.99)		4.04 (1.54)	
The employment specialist seemed professional (based on PSO-HP)	4.26 (1.16)		4.08 (1.29)		3.84 (1.31)		3.84 (1.39)		4.32 (1.10)		4.38 (1.03)		4.38 (1.01)		4.00 (1.38)	
Overall, I am satisfied with the guidance of the employment specialist (based on PSO-HP)	4.12 (1.39)		3.92 (1.45)		3.72 (1.45)		3.81 (1.35)		4.26 (1.10)		4.27 (1.19)		4.36 (1.12)		3.88 (1.48)	
Questions only answered by people who did not have a job at that measurement; (1 = totally disagree, 5 = totally agree)																
Return to work self-efficacy (RTW-SE)	3.84 (1.14)		3.87 (1.13)		3.8 (1.17)		3.7 (1.10)		4.1 (1.03)		4.06 (0.96)		3.76 (1.11)		3.73 (0.94)	
I feel stressed to find a job	3.37 (1.48)		3.14 (1.46)		3.34 (1.38)		3.24 (1.23)		3.45 (1.41)		3.41 (1.44)		3.28 (1.30)		3.18 (1.36)	
It is exciting to find a job	3.91 (1.05)		3.59 (1.36)		3.68 (1.13)		3.62 (0.88)		3.87 (1.07)		3.65 (1.13)		3.82 (1.17)		3.52 (1.35)	
I will succeed to find a job	3.69 (1.21)		3.29 (1.33)		3.28 (1.26)		3.14 (1.05)		3.87 (0.92)		3.67 (1.11)		3.55 (1.08)		3.27 (0.98)	
Because of my MHP, I have less opportunities to find a job	3.09 (1.33)		3.16 (1.38)		3.34 (1.27)		3.12 (1.21)		2.96 (1.30)		2.96 (1.24)		3.1 (1.25)		2.85 (1.39)	
I think my MHP affect my job performance	3.3 (1.32)		3.29 (1.20)		3.2 (1.40)		3.21 (1.24)		3.07 (1.27)		3.04 (1.15)		3.18 (1.14)		3 (1.25)	

## Discussion

The findings of the current study show that the stigma awareness intervention was highly effective in improving work participation outcomes; six months after baseline, significantly more participants of the experimental group had *found* paid employment compared to the control group (50.7% versus 26.1%). Moreover, twelve months after baseline, significantly more participants of the experimental group had *retained* paid employment compared to the control group (49.2% versus 23.4%). The intervention had no effect on decisional conflict and stage of decision making. Interestingly, six months after baseline, in the experimental group participants were significantly more positive about the support received from their employment specialists.

This study adds to the growing evidence that stigma and discrimination contributes to lower employment rates of people with MHI, and cannot solely be attributed to their MHI. Our trial showed that the decisional process concerning communication about MHI, rather than the actual illness itself, largely determined if people found and retained paid work. The disclosure process therefore is of key importance for reemployment success. This is in line with conclusions of others [10, 15–17], e.g. Rusch and colleagues who found that greater reluctance to disclose mental health problems among the unemployed, predicted finding employment more often 6 months later [15]. An important new insight from the present study, is that in this study population, this disclosure process could successfully be influenced by the intervention, resulting in higher and more sustainable employment rates of our study population. As the percentage of people who found and retained paid work almost doubled, this suggests that on a societal level, a vast number of unemployed people could be reemployed with a relatively simple intervention, potentially leading to increased health and recovery, and major savings on social benefits.

In contrast to earlier studies [16, 20], no effects on decisional conflict and stage of decision making were found. This might be explained by the differences in selection criteria. The current study did not use cut off scores for selecting people having at least moderate decisional stress, like the earlier study by Henderson and colleagues [16]. Alternatively, this could be explained by cultural and legal differences. In particular, it is known that most Dutch people with MHI have preferences to disclose their MHI to their employer [18]. Possibly, this is related to the highly protective Dutch legislation for employees, including legislation to protect sick listed employees, financial subsidies for employees with disabilities and financial obligations for employers when an employee becomes sick [45].

The stigma awareness training for employment specialists is one of the two key elements of the intervention. Employment specialists are important stakeholders for the employment opportunities of unemployed people with MHI and therefore it is important that they give them the right support. However, because of their mediating role between unemployed people and employers, employment specialists might prefer disclosure of MHI to not harm the professional relationships with employers [17]. Increasing awareness amongst employment specialists about stigma and discrimination in the work environment (e.g. employers are reluctant to hire employees who have (had) MHI [13, 17]), and giving insight into the effects of one’s personal attitudes, prejudices and actions may have improved the quality of the vocational rehabilitation services. Moreover, participants in the experimental group reported a higher quality of support by their employment specialist. This suggests that potentially, due to the stigma awareness training, employment specialists gained more understanding and improved their skills in having conversations about the impact of MHI and its consequences about whether to disclose this or not. Subsequently they could have delivered better vocational guidance to people with MHI. Although these seem plausible explanations for the reported effect of the intervention, especially for long term effects, more insight is needed into the working elements of complex interventions and what works for whom [46].

A key strength of this study is the randomised controlled design with clusters at practice level, which prevents contamination between individual unemployed participants and their employment specialists. Another strength is the use of several measurements over 12 months, during which large efforts were taken to prevent drop out of participants, resulting in lower dropout rates than expected. A limitation of this study is that, although the exact intervention was not known by employment specialists of the control group, employment specialists of both conditions were aware that they were participating in a study on improving work participation outcomes of people with MHI. This awareness may have altered the behaviour of both groups, i.e. employment specialists of both groups could have become more motivated to support people with MHI, which is also known as the Hawthorne Effect [47]. In addition, participants were recruited via employment specialists. This may have caused selection bias e.g. because employment specialists may not always be aware of the (history of) MHI of some of his clients, or because they judge some of their clients as not eligible or capable to participate in the study. Other limitations of the study is the lack of involvement of employers in this intervention, as they are important stakeholders [12], and the use of self-report data only.

Future research should focus on the effects of the intervention implemented in existing evidence-based practices to improve employment outcomes. As this is one of the first intervention studies on the effects of stigma on sustainable employment for people with MHI, more research into the effectiveness and the working elements is needed. In this study, it is not known if participants switched between jobs during the study. However, as people with MHI are 3 to 7 times more likely to be unemployed than people without MHI [1], finding and retaining employment is an outstanding achievement. As 49% of the participants retained paid employment after twelve months, we suggest that there is a suitable person-job fit in most cases because people had a sustainable form of employment. Furthermore, as also people with other health problems, e.g. physical disabilities or illnesses, experience stigma [12], it is likely that those people will face similar challenges while finding or retaining work. Therefore, it may be useful to adapt the intervention also to other health problems. However, more research on this area is needed.

In conclusion, this study showed that six months after baseline, about twice as many participants of the experimental group had *found* paid employment compared to the control group and twelve months after baseline, about twice as many of those in the experimental group had *retained* paid employment. These findings underline the importance of research on destigmatizing interventions. Moreover, they suggest that on a societal level, a vast number of unemployed people could be reemployed with a relatively simple intervention, potentially leading to increased health and recovery, and major savings on social benefits.

### Supplementary Information

Below is the link to the electronic supplementary material.

Supplementary file1 (PDF 244 KB) CORAL decision aid (EN version)

Supplementary file2 (PDF 399 KB) CORAL decision aid (Dutch version)

Supplementary file3 (PDF 300 KB) Infographic about disclosure during job interviews (in Dutch)

Supplementary file4 (PDF 299 KB) Infographic about disclosure at work (in Dutch)

Supplementary file5 (DOCX 19 KB) Stage of decision making

## Data Availability

The datasets generated during and/or analysed during the current study are available from the corresponding author on reasonable request.

## Abbreviations

RCT: Randomized controlled trial
CORAL: Conceal or reveal
MHI: Mental health issues/mental illness
