# Supplementary material for: Effectiveness of a Stigma Awareness Intervention on Reemployment of People with Mental Health Issues/Mental Illness: A Cluster Randomised Controlled Trial
Source: J Occup Rehabil. 2023 Jul 13;34(1):87–99. doi: 10.1007/s10926-023-10129-z (PMC10899371; doi:10.1007/s10926-023-10129-z)
Supplement: Supplementary file 1 — Supplementary file1 (PDF 244 KB) CORAL decision aid (EN version) [file 10926_2023_10129_MOESM1_ESM.pdf]

Conceal or Reveal: A guide to telling employers about a mental health condition (V3:30.11.15)

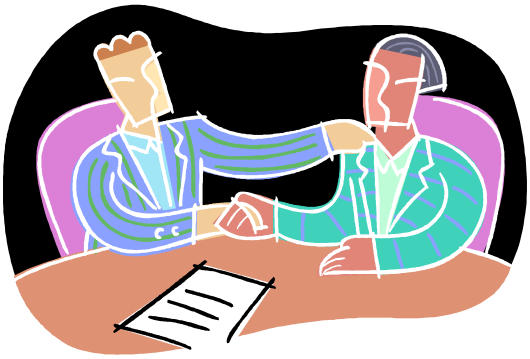

Who is this booklet for?

This booklet is for anyone who has experienced a mental health condition and is unsure of issues related to telling a potential or current employer about this.

Where did the information in this booklet come from?

The information in this booklet is based on interviews with people who have a mental health condition and a review of published literature in this area. Throughout the booklet we will use the term 'disclosure' to mean telling an employer about a mental health condition.

Is there a right decision?

This guide aims to help you find the decision that is right for you by considering your needs and values. It also helps you to think about when to disclose and who to disclose to.

Who can I contact for information about the booklet?

If you would like further information about the booklet then please contact:

Dr Claire Henderson
Section of Community Mental Health Box PO29
Health Service and Population Research Department
King's College London Institute of Psychiatry,
De Crespigny Park,
London SE5 8AF
(Telephone) +44 (0)20 7848 0570
(Email) claire.1.henderson@kcl.ac.uk

© King's College London
This document should not be modified in any way or translated without permission from Dr Henderson (contact details above)

KING'S College LONDON

University of London

1. Pros and cons of disclosure

This first section provides a list of the pros and cons of disclosure. This list includes lots of possible outcomes so you can consider which would be most important to you.

Pros of disclosure

- ☑ Disability discrimination legislation requires that employers make 'reasonable adjustments' that a person who is disabled by a mental health condition may need to do the job (e.g. adjustments in hours or parts of the job or working conditions). Employers are only required to make these if they are aware of your condition
- ☑ You may not have to hide any difficulties you have – and may be able to ask for help, time off or a reduced work load at times when you are having difficulties
- ☑ You can ask your employer for time off to go to things like doctor's appointments
- ☑ You can be honest with your colleagues – it can be very difficult hiding a 'big secret' all of the time
- ☑ If you have an employment advisor or occupational therapist then disclosure can allow them to liaise directly with the employer to provide support around reasonable adjustments
- ☑ If you don't tell your colleagues and they find out they may gossip behind your back
- ☑ If you tell people about your condition then you will be helping other people with similar difficulties. If your employer can see you are able to do the job the job they may be more likely to employ someone else with a mental health condition. You can help break down some of the prejudiced attitudes of your colleagues and enable others who have similar difficulties to talk about them

Cons of disclosure

- ☒ You may be less likely to get the job
- ☒ If you have already got a job, you may be less likely to get a promotion
- ☒ You may be sacked if you do not disclose your mental health condition if asked during the recruitment process and it is found out about later
- ☒ Your employer may not trust you with responsible jobs
- ☒ If you ask for help with something at work they may think that you are not able to do the work because of your mental health condition
- ☒ You may have to be twice as good as anyone else to prove that you can do the job
- ☒ Every time you have a bad day, or get cross, or upset, they may think that this is because of your mental health condition and conclude that you are not up to the job (even if the problems you are experiencing are perfectly ordinary difficulties that might affect the work of anyone)
- ☒ Your colleagues may treat you differently if they know you have a mental health condition – they may be awkward with you, gossip about you behind your back, not want to be friends with you, not trust you

Adapted from: Department for Work and Pensions. (2009). Realising Ambitions: Better employment support for people with a mental health condition.

To think more about the pros and cons, it may help to write down the three pros and cons which are most important to you. You can refer back to this box as you make your decision at the end of the booklet.

For me the main pros of disclosure are:
1.____
2.____
3.____

For me the main cons of disclosure are:
1.____
2.____
3.____

2. Disclosure needs

This section focuses on helping you to identify your disclosure needs. A ‘need’ is something which it is important for you to have in order to do your job and feel satisfied at work. These are different things for different people. Some people decide to disclose because this allows them to meet certain needs.
Below are some examples of needs described by the people we interviewed:

‘If they asked me why I wanted to work part time I probably would have to tell them if maybe if they tried to offer me full time then I would have to say this is reason I want to work part time, but I would rather not tell them, just not to hinder my chances really’ [Jeffrey]

‘I mean obviously if you had loads of appointments and stuff to keep then yeah you know you would have to say something and then you would benefit from it, you know, they would let you have the time off and stuff but I don’t see that it would benefit normally’ [Tina]

‘I would say if you have a mental illness, depending on what your symptoms and your diagnosis is and how well you are, it’s down to you as the individual, if you feel that you need to discuss it’ [Daniel]

‘I’m not going to lie. At the end of the day, I won’t keep a job if I get a job and I am there a week and then all of a sudden it hits me again and then I can’t go in for that week ‘cos if I’m going in there I’m going to be in tears, I’m visibly crying sometimes yeah, I am not going to cry in front of everybody, not speak to anybody, not be able to do my job’ [John]

‘I don’t know… say if I’m not coping too well and finding it a bit hard, I might get more help if like I was honest, instead of like oh this is the problem and I might need a bit of extra help in certain areas so yeah I think it would help me’ [Sharon]

Thinking about your workplace needs can be a useful way to clarify whether disclosure is right for you. Below is a list of different needs which may influence disclosure. Please read the list and tick any statements which you agree with:

- ☐ I need to make changes to my working hours because of my mental health condition
- ☐ I need to attend medical appointments during work hours because of my mental health condition
- ☐ I may need to take time off because of my mental health condition
- ☐ My mental health condition means that I may have difficulty in performing certain aspects of the job
- ☐ I need adjustments in the workplace to perform effectively in my job
- ☐ I experience symptoms of illness or side effects of medication which may make my mental health condition noticeable to others
- ☐ I have gaps in my CV due to a mental health condition
- ☐ I work in a job where having personal experience of a mental health condition may be an advantage
- ☐ I need to declare my mental health condition as I have been asked on a job application form
- ☐ I need to have a plan in place with my employer about what to do if I become ill at work or need to take time off

To think more about what you need in the workplace, it may help to write down the three things which are most important to you. You can refer back to this box as you make your decision at the end of the booklet.

For me the things I need in the workplace are:
1.____
2.____
3.____

3. Disclosure values

This section describes values. A value is a trait or quality which a person considers worthwhile. They describe how you would like to behave and the things which are of greatest importance to you. For some people disclosure decisions are made by thinking about what best fits with their values. Below are some examples of values reported by the people we interviewed:

'Just not to be treated differently, that was the main thing you know, I just wanted to be treated like everyone else who worked there and as long as it's not causing any problem with the work you know then I don't see the need, I've had loads of jobs where I haven't said anything to anyone, nobody knows. It's just to be treated the same as everyone else really' [Tina]

'It's kind of an individual thing, it's kind of how you would react to somebody else laughing at you about it, people talking about you behind your back, it's how you would handle that and I think it's very much an individual thing' [Tina]

'I was very successful because at the end of the day as you've probably guessed, I am not afraid to speak to people about illness, at the end of the day by doing that I find I get any of the jobs' [John]

'How one feels and how one is confident with his illness or her illness is for him or her to decide, not someone else' [Imran]

"I wouldn't be able to feel comfortable walking away saying no, those declarations, and you have to sign them, so I think no. I mean at the end of the day if I don't have a clear conscience, I would rather have that than if you have work and find out I have some disturbance' [Michael]

'I've never hidden my illness from anyone, I'm not ashamed of it, I'm not ashamed of it, you know some people they do treat you differently' [Sharon]

Below is a list of different values related to disclosure decisions. Please read the list and tick any statements which you agree with:

- ☐ I value being open and honest about my mental health condition
- ☐ I value keeping my mental health condition a private part of my life
- ☐ I value being treated the same as anybody else in the workplace
- ☐ I value receiving support and reasonable adjustments in the workplace
- ☐ I would like to be a role model for other people with mental health conditions
- ☐ I do not want to share my mental health condition with others
- ☐ I find it important to challenge negative attitudes to mental health conditions when I hear them
- ☐ I prefer to remove myself from situations where people have negative attitudes to mental health conditions
- ☐ I find it difficult or upsetting to deal with negative attitudes to mental health conditions
- ☐ I would like to educate my employer and colleagues about what it means to have a mental health condition
- ☐ I want to avoid reactions such as being pitied, patronised, rejected or gossiped about
- ☐ I want to be myself at work even if this mean people may disapprove or treat me differently
- ☐ I would find hiding my mental health condition very stressful
- ☐ I would find talking about my mental health condition very stressful

To think more about what you value in the workplace, it may help to write down the three things which are most important to you. You can refer back to this box as you make your decision at the end of the booklet.

For me the things I value in the workplace are:
1.____
2.____
3.____

4. When to disclose?

As well as deciding whether to disclosure, it is useful to focus on when to disclose. Below are examples of different times to disclose reported by the people we interviewed:

‘Yes I always state it on my cv ‘cos I don’t feel that dishonesty is the way forward’ [Dave]

‘I finally told my managers when I had an extended period of depression that lasted for 5 months and I had to take time off work, this was last year, so I had to tell them, I wouldn’t have if that situation wouldn’t have come up’ [Alison]

‘I don’t want people to form their opinions of me without seeing for themselves the facts first of what I am actually like, I mean it’s the old saying that you can’t judge a book by its cover, do you know what I mean, you’ve got to get to know the person first and see for yourself, not just to form an opinion on nothing. It’s not fair to me or to anybody else, check your facts first then make your mind up and be more open minded, that’s what I say’ [Sharon]

‘I think before they can say anything you’ve got to educate your employer basically on what that actually means…if they have met you, you’ve got the job and then you told them a few months down the line then I don’t think it would be such a big deal but yeah I think it stops you from getting a job’ [Tina]

The table on the next page presents the advantages and disadvantages of disclosure at various points in the employment journey. Please read through this table and tick the box below which you feel fits with your views on the timing of disclosure:

For me the best time to disclose is (tick one):
- ☐ On job application form
- ☐ During an interview
- ☐ After interview, if offered a job (before taking up job)
- ☐ After the interview at occupational health assessment
- ☐ Once in the job
- ☐ At a later stage
- ☐ Never

Time of disclosure	Advantages	Disadvantages
1. On a job application	☑ Honesty/Peace of Mind<br>☑ Easy. Lets employer decide if your mental health condition is an issue	☒ Might disqualify you with no opportunity to present yourself and your qualifications<br>☒ Potential for discrimination
2. During an interview	☑ Honesty/Peace of mind<br>☑ Opportunity to respond briefly and positively – in person – to specific issues about mental health condition<br>☑ Discrimination may be less likely face-to-face	☒ Puts responsibility on you to handle questions about mental health condition issues in a clear, non-threatening way.<br>☒ Too much emphasis may be placed on the condition rather than your abilities
3. After the interview (when a job is offered but before you begin work)	☑ Honesty/Peace of mind<br>☑ If the disability changes the hiring decision and you are sure that your limitations will not interfere with your ability to perform the job, there may be legal recourse<br>☑ Allows you to ask for reasonable adjustments if you need this	☒ Employer might feel you should have told her/him before hiring decision was made<br>☒ Might lead to distrust with personnel department
4. After you start work/at a later stage/never	☑ Opportunity to prove yourself on job before disclosure<br>☑ People get to know other aspect of your personality and see you as a whole person<br>☑ You get to know your employer and colleagues and judge whether they would react well and who to	☒ Nervousness or fear of having an episode/accident on the job<br>☒ Possible employer accusation of falsifying your application<br>☒ Possibility of an episode/accident before co-workers know how to react<br>☒ Could change interaction with peers

Adapted from: Mental Health Council of Australia. (2007). Let's get to work: A national mental health strategy for Australia.

5. Who to disclose to?

The next thing to think about is who to tell. Some people prefer not to tell anyone, others prefer to tell only their employer or line manager, others would also like certain colleagues to know whereas some like to be completely open about their mental health condition and have it as something which everybody knows about. Below are examples of different people to disclose to reported by the people we interviewed:

'You know there are some people you can work for and if you tell them that you have suffered with mental health or you have a mental health illness or whatever, some employers will be supportive but others might look at that as a risk factor you know. [Daniel]

'I don't know if I would want all my work colleagues to know that I have a mental condition. I don't know, my friends know, but I don't know if I would want work colleagues to know' [Jeffrey]

'I've never hidden my illness from anyone, I'm not ashamed of it, I'm not ashamed of it, you know some people they do treat you differently' [Sharon]

'I don't think it's the employers, but it's the people you work with for example, if they hear it or find out about that particular, he suffers from illness, they would look down upon you, its human nature to do that' [Imran]

The table on the next page presents the advantages and disadvantages of telling various people about your mental health condition. Please read through this table and tick the box below which you feel best fits with your views regarding who to disclose to:

The disclosure strategy which would work best for me is (tick one):<br><br>☐ Secrecy<br>☐ Selective disclosure<br>☐ Indiscriminate disclosure<br>☐ Broadcast my experience

	Pros	Cons
**1. Secrecy**: Don't tell anybody at work about your mental health condition	☑ Nobody can use this information to hurt you<br>☑ You are treated the same as everybody else	☒ Some people feel anxious or guilty about keeping secrets<br>☒ You cannot gain reasonable adjustments without disclosing<br>☒ May lose your job if it is found out
**2. Selective disclosure**: Tell some people who you believe will be supportive about your mental health condition	☑ You find a small group of people who will understand your experiences and provide support. This can include employer or colleagues<br>☑ You can gain reasonable adjustments	☒ You may disclose to some people who will not react well or who may hurt you with the information<br>☒ You may have difficulty keeping track of who knows and who doesn't
**3. Indiscriminate disclosure**: Don't be concerned about who knows about your mental illness. Tell anyone you encounter	☑ You don't worry about who knows about your condition.<br>☑ You are likely to find some people who will be supportive	☒ You are likely to disclose to some people who will not react well or who will hurt you with the information
**4. Broadcast your experience**: Purposefully communicate your experiences to a large group e.g. making an announcement to the whole organisation or at a staff meeting	☑ You don't have to worry who knows about your mental health condition.<br>☑ You are promoting a personal sense of empowerment in yourself<br>☑ May help other people to disclose<br>☑ You are helping to combat stigma.	☒ You are going to encounter people who react badly or try to hurt you with this information.<br>☒ You may find that you are frequently called upon to give your opinion on mental health issues and need to be comfortable with this identity

Adapted From: Corrigan, P.W. & Lundin, R. (2001). Don't Call Me Nuts!: Coping with the Stigma of Mental Illness. Tinley Park, IL: Recovery Press.

6. Making a decision

At this stage you will have worked through the booklet and considered each of the below:

1. The pros and cons of disclosure
2. My disclosure needs
3. My disclosure values
4. When to tell
5. Who to tell

By looking back over your answers in each of the 5 pink boxes for each section you will be more aware of the issues related to your disclosure decision. This may be something which you would like to discuss with family, friends or a mental health professional to talk through any outstanding issues.

On the next page you can write what you plan to say when disclosing. Or, if you plan not to disclose, you can write what to say if you need to say something *instead* of disclosing. For example, you might be asked about a gap in your CV.

You may not feel you are in a position to make a decision yet. In this case we suggest you write both what you would say when disclosing and what you would say instead of disclosing.

My decision about when to disclose is:

My decision about whom to disclose to is:

If I disclose I am going to say the following:

If I have the opportunity to disclose and do not want to take it, I will instead say:

I would like to discuss this decision with:
