# Supplementary material for: Effectiveness of a Stigma Awareness Intervention on Reemployment of People with Mental Health Issues/Mental Illness: A Cluster Randomised Controlled Trial
Source: J Occup Rehabil. 2023 Jul 13;34(1):87–99. doi: 10.1007/s10926-023-10129-z (PMC10899371; doi:10.1007/s10926-023-10129-z)
Supplement: Supplementary file 2 — Supplementary file2 (PDF 399 KB) CORAL decision aid (Dutch version) [file 10926_2023_10129_MOESM3_ESM.pdf]

Psychische gezondheidsproblemen

Veel mensen die psychische problemen hebben (gehad), vragen zich af of het verstandig is om dit te vertellen tijdens een sollicitatie.

Hebt u hier al over nagedacht?

Belangrijke redenen om <u>niet</u> over uw psychische problemen te vertellen tijdens een sollicitatie:

Er zijn veel vooroordelen, waardoor u mogelijk

- Niet aangenomen wordt.
- Een korter contract, lager salaris of geen vast contract krijgt.
- Anders (negatiever) behandeld wordt.

Belangrijke redenen om <u>wel</u> over uw psychische problemen te vertellen tijdens een sollicitatie:

- Uw werkgever kan beter rekening houden met wat u nodig heeft.
- U kunt beter toelichten waarom u bijvoorbeeld periodes niet heeft gewerkt (gaten in uw CV) of langer heeft gestudeerd.
- Als u zich hier veel prettiger bij voelt of als u zo meer uzelf kan zijn.

Tips

- De sollicitatieperiode is vaak minder geschikt om open te zijn over psychische gezondheidsproblemen, omdat mensen u dan nog niet goed kennen. Het is vaak beter om eerst te laten zien wie u bent en wat u kunt.
- Voor uw werkgever is het belangrijk om te weten wat u nodig hebt om uw werk goed te doen.
- Uw werkgever hoeft niet alles van u te weten, het is normaal dat sommige dingen privé zijn.

Tilburg University
School of Social and Behavioral Sciences

Meer weten?
Lees dan de brochure
'CORAL.NL'.

Tranzo
Wetenschappelijk centrum voor zorg en welzijn
