# Supplementary material for: Effectiveness of a Stigma Awareness Intervention on Reemployment of People with Mental Health Issues/Mental Illness: A Cluster Randomised Controlled Trial
Source: J Occup Rehabil. 2023 Jul 13;34(1):87–99. doi: 10.1007/s10926-023-10129-z (PMC10899371; doi:10.1007/s10926-023-10129-z)
Supplement: Supplementary file 3 — Supplementary file3 (PDF 300 KB) Infographic about disclosure during job interviews (in Dutch) [file 10926_2023_10129_MOESM4_ESM.pdf]

Psychische gezondheidsproblemen

Veel mensen die psychische problemen hebben (gehad), vragen zich af of het verstandig is om dit te vertellen op het werk.

Hebt u hier al over nagedacht?

Belangrijke redenen om niet over uw psychische problemen te vertellen op het werk:

Er zijn veel vooroordelen, waardoor u mogelijk:

- Geen contractverlenging krijgt.
- Geen promotie maakt omdat u lager wordt ingeschat.
- Anders (negatiever) behandeld wordt.

Belangrijke redenen om wel over uw psychische problemen te vertellen op het werk:

- Uw werkgever kan beter rekening houden met wat u nodig heeft.
- Openheid kan leiden tot meer begrip voor u en uw situatie.
- U kunt mogelijk meer uzelf zijn.
- Als u open bent zullen anderen zich ook sneller openstellen en persoonlijke dingen durven te vertellen.

Tips

- Als uw psychische problemen niet van invloed zijn op het werk is het belangrijk goed na te denken of u het wel wilt vertellen.
- Als u open wilt zijn, vertelt u dan vooral wat u nodig heeft om uw werk goed te doen en leg minder de nadruk op privézaken of medische dingen.

Meer weten?
Lees dan de brochure
'CORAL.NL'.
