# Supplementary material for: Effectiveness of a Stigma Awareness Intervention on Reemployment of People with Mental Health Issues/Mental Illness: A Cluster Randomised Controlled Trial
Source: J Occup Rehabil. 2023 Jul 13;34(1):87–99. doi: 10.1007/s10926-023-10129-z (PMC10899371; doi:10.1007/s10926-023-10129-z)
Supplement: Supplementary file 4 — Supplementary file4 (PDF 299 KB) Infographic about disclosure at work (in Dutch) [file 10926_2023_10129_MOESM5_ESM.docx]

Manuscript

**Effectiveness of a stigma awareness intervention on reemployment of people with mental health issues/mental illness: a cluster randomised controlled trial**

| **Appendix 2.** Primary outcomes – stage of decision making. | | | | | | | | | |
| --- | --- | --- | --- | --- | --- | --- | --- | --- | --- |
|  |  |  |  | **Control group** |  | **Experimental group** |  | **OR (95% CI)** | **P-value** |
|  |  |  |  | **N (%)** |  | **N (%)** |  |  |  |
| Stage of decision making | | |  |  |  |  |  |  |  |
|  | T0 | Haven’t begun to think about the choices |  | 11 (14.3) |  | 11 (14.7) |  |  |  |
|  |  | Haven’t begun to think about the choices, but is interested in doing so |  | 7 (9.1) |  | 4 (5.3) |  |  |  |
|  |  | Are considering the options now |  | 9 (11.7) |  | 9 (12) |  |  |  |
|  |  | Are close to selecting an option |  | 1 (1.3) |  | 5 (6.7) |  |  |  |
|  |  | Have already made a decision, but is still willing to reconsider |  | 22 (28.6) |  | 19 (25.3) |  |  |  |
|  |  | Have already made a decision and is unlikely to change my mind |  | 27 (35.1) |  | 27 (36) |  |  |  |
|  | T1 | Haven’t begun to think about the choices |  | 7 (10.1) |  | 10 (14.9) |  | 0.61 (0.29 - 1.28) | 0.19 |
|  |  | Haven’t begun to think about the choices, but is interested in doing so |  | 7 (10.1) |  | 5 (7.5) |  |  |  |
|  |  | Are considering the options now |  | 8 (11.6) |  | 10 (14.9) |  |  |  |
|  |  | Are close to selecting an option |  | 3 (4.3) |  | 1 (1.5) |  |  |  |
|  |  | Have already made a decision, but is still willing to reconsider |  | 13 (18.8) |  | 19 (28.4) |  |  |  |
|  |  | Have already made a decision and is unlikely to change my mind |  | 31 (44.9) |  | 22 (32.8) |  |  |  |
|  | T2 | Haven’t begun to think about the choices |  | 8 (11.6) |  | 10 (14.9) |  | 1.15 (0.54 - 2.42) | 0.72 |
|  |  | Haven’t begun to think about the choices, but is interested in doing so |  | 5 (7.2) |  | 2 (3) |  |  |  |
|  |  | Are considering the options now |  | 6 (8.7) |  | 6 (9) |  |  |  |
|  |  | Are close to selecting an option |  | 5 (7.2) |  | 1 (1.5) |  |  |  |
|  |  | Have already made a decision, but is still willing to reconsider |  | 19 (27.5) |  | 19 (28.4) |  |  |  |
|  |  | Have already made a decision and is unlikely to change my mind |  | 26 (37.7) |  | 29 (43.3) |  |  |  |
|  | T3 | Haven’t begun to think about the choices |  | 13 (20.3) |  | 11 (17.2) |  | 1.24 (0.57 - 2.7) | 0.59 |
|  |  | Haven’t begun to think about the choices, but is interested in doing so |  | 4 (6.2) |  | 4 (6.2) |  |  |  |
|  |  | Are considering the options now |  | 5 (7.8) |  | 2 (3.1) |  |  |  |
|  |  | Are close to selecting an option |  | 1 (1.6) |  | 5 (7.8) |  |  |  |
|  |  | Have already made a decision, but is still willing to reconsider |  | 16 (25) |  | 16 (25) |  |  |  |
|  |  | Have already made a decision and is unlikely to change my mind |  | 25 (39.1) |  | 26 (40.6) |  |  |  |
