# Supplementary material for: Effectiveness of a Stigma Awareness Intervention on Reemployment of People with Mental Health Issues/Mental Illness: A Cluster Randomised Controlled Trial
Source: J Occup Rehabil. 2023 Jul 13;34(1):87–99. doi: 10.1007/s10926-023-10129-z (PMC10899371; doi:10.1007/s10926-023-10129-z)
Supplement: Supplementary file 5 — Supplementary file5 (DOCX 19 KB) Stage of decision making [file 10926_2023_10129_MOESM2_ESM.pdf]

CORAL.NL
Afwegingen rond openheid over psychische gezondheidsproblemen op het werk: Een keuzehulp

Colofon

Titel: CORAL.NL. Afwegingen rond openheid over psychische gezondheidsproblemen op het werk: Een keuzehulp.
Oorspronkelijke titel: CORAL: Conceal or Reveal
Auteur: Dr. Claire Henderson, King's College London

Deze keuzehulp is een aangepaste versie van de Nederlandse vertaling 'Verzwijgen of Vertellen'. Deze versie is een uitgave van Samen Sterk Zonder Stigma en is tot stand gekomen in samenwerking tussen Kenniscentrum Phrenos, Tranzo/Tilburg University en Samen Sterk Zonder Stigma.

Contactpersoon: Kim Janssens, e: k.m.e.janssens@tilburguniversity.edu, t: +31 13 466 4696

Meer informatie over de CORAL: Dorien Verhoeven, e: d.verhoeven@samensterkzonderstigma.nl

SAMEN STERK ZONDER STIGMA

Phrenos kenniscentrum

Tranzo Wetenschappelijk centrum voor zorg en welzijn

TILBURG UNIVERSITY School of Social and Behavioral Sciences

CORAL.NL
Afwegingen rond openheid over psychische gezondheidsproblemen op het werk: Een keuzehulp

Wat is het doel van deze keuzehulp?

Kun je wel of niet open zijn over psychische gezondheidsproblemen[1] op je werk, of als je gaat solliciteren? Wat is de meest verstandige keuze? Daarover twijfelen veel mensen. Deze vragen kunnen vaker terugkomen en het kan per situatie verschillen wat verstandig is. Het doel van deze keuzehulp[2] is om mensen die hierover nadenken te ondersteunen. De keuze ligt altijd bij de persoon zelf. Door het lezen van de overwegingen in deze keuzehulp kunnen mensen op nieuwe ideeën komen die helpen bij het maken van eigen keuzes. Ook biedt het handvatten bij het ondersteunen van anderen die voor deze keuzes staan.

Voor wie is deze keuzehulp?

Deze keuzehulp is voor iedereen die psychische gezondheidsproblemen heeft of heeft gehad en nadenkt over wat hij/zij hierover wel en niet aan een huidige of toekomstige werkgever of anderen in de werkomgeving wil vertellen. Daarnaast kunnen professionals, op het gebied van bijvoorbeeld arbeidsre-integratie, en werkgevers deze keuzehulp inzetten. Ook naasten van iemand die vragen heeft over het bespreekbaar maken van psychische gezondheidsproblemen, kunnen baat hebben bij deze keuzehulp.

Inhoud

In het eerste deel van deze keuzehulp komen argumenten aan bod die een rol kunnen spelen bij open of niet open zijn in de werkomgeving (Deel 1: Keuzes rond openheid). Hier gaat het erom inzicht te krijgen in wat je nodig hebt en belangrijk vindt. In deel 2 word je uitgenodigd na te denken over of je wel of niet open wilt zijn. En zo ja, wanneer en met wie je in gesprek zou willen gaan. (Deel 2: Mijn eigen situatie). Deel 3 geeft nog een aantal tips, die je verder kunnen helpen bij je keuzes. Op basis van je persoonlijke afwegingen kun je in deel 4 aangeven wat op dit moment je keuzes (zouden) zijn rond het bespreekbaar maken van psychische gezondheidsproblemen in de werkomgeving (Deel 4: Een keuze maken).

---

[1] Waar we het hebben over psychische gezondheidsproblemen kan ook gedacht worden aan psychische aandoeningen/stoornissen/kwetsbaarheid/gevoeligheid. Je kunt afwegen welke term je wilt gebruiken wanneer je het hebt over je psychische gezondheid.

[2] Deze keuzehulp is onderdeel van een module die tevens een programma voor een startbijeenkomst en intervisiebijeenkomsten voor professionals, en een handleiding voor professionals bevat.

DEEL 1: Keuzes rond openheid

1.1 Argumenten

Er zijn verschillende argumenten te bedenken om wel of niet open te zijn over psychische gezondheidsproblemen in de werkomgeving of bij een sollicitatie. Hieronder vind je eerst een aantal argumenten voor openheid en daarna een aantal argumenten tegen.

Je kunt aanvinken welke hiervan voor jou gelden en het meest belangrijk of relevant voor je zijn.

Argumenten om open te zijn over psychische gezondheidsproblemen in de werkomgeving

- ❑ Als je niets hoeft te verzwijgen, is dat goed voor je zelfvertrouwen.
- ❑ Je kunt begrip en steun krijgen van je leidinggevende of collega's.
- ❑ Het kan voor jou als (toekomstige) werknemer en voor je werkgever fijn zijn om je psychische gezondheid te bespreken, vooral als er aanpassingen op je werk nodig zijn. Er kan rekening mee gehouden worden.
- ❑ Het kan voor problemen zorgen als anderen er later achter komen.
- ❑ Het kan moeilijk en stressvol zijn om met een 'geheim' rond te lopen.
- ❑ Het kan zijn dat men al wist of vermoedde dat er iets aan de hand was.
- ❑ Door eerlijk te zijn tegen anderen op je werk laat je zien dat je hen vertrouwt. Contacten kunnen verbeteren en je weet wat je aan elkaar hebt.
- ❑ Als je het niet vertelt, en mensen merken iets aan je, dan zouden ze dat op een bepaalde manier kunnen gaan invullen, waardoor het misschien erger wordt ingeschat dan wanneer je open bent.
- ❑ Je kunt het onderwerp psychische gezondheid meer bespreekbaar maken en het makkelijker maken voor anderen om over hun psychische gezondheid te praten.
- ❑ Als je werkgever ziet dat jij je werk kan doen, is de kans groter dat hij/zij nog iemand aanneemt met psychische gezondheidsproblemen.
- ❑ Het kan zijn dat je een gat in je CV hebt als gevolg van gezondheidsproblemen en dit wilt kunnen toelichten.
- ❑ Het kan zijn dat je een baan hebt of wilt, waarbij het in je voordeel zou kunnen werken dat je zelf psychische gezondheidsproblemen hebt (gehad).
- ❑ Door open te zijn kun je ook meer van je (andere) positieve eigenschappen laten zien.
- ❑ Een extra argument voor openheid is volgens mij:
  ____

Argumenten om niet open over psychische gezondheidsproblemen te zijn in de werkomgeving

- ❑ Het is niet van invloed op hoe je je werk doet.
- ❑ Je wilt geen onnodig risico lopen op negatieve reacties.
- ❑ Je wordt misschien niet aangenomen als je vertelt over je psychische gezondheidsproblemen in een sollicitatie- of arbeidsvoorwaardengesprek.
- ❑ Je (toekomstige) werkgever kan bang zijn dat je gaat uitvallen en te veel kijken naar waar het misgaat.
- ❑ Je kunt het gevoel krijgen dat je op je tenen moet gaan lopen als je erover zou vertellen.
- ❑ Je kunt je onprettig voelen als bepaalde informatie bij je werkgever bekend is.
- ❑ Erover vertellen kan de kans op promotie of een verlenging van je contract verminderen.
- ❑ Je werkgever zal je bepaalde verantwoordelijkheden misschien niet toevertrouwen.
- ❑ Je leidinggevende of collega's zouden kunnen denken dat je vanwege je gezondheidsproblemen niet in staat bent je werk (goed) te doen.
- ❑ Als je niet lekker in je vel zit of als je een slechte dag hebt, zouden ze kunnen denken dat dit te ermee te maken heeft en de conclusie trekken dat je niet geschikt bent voor dit soort werk (zelfs als het alledaagse problemen zijn die ieders werk zouden beïnvloeden).
- ❑ Als je collega's het weten, zouden ze je anders kunnen behandelen.
- ❑ Je collega's zouden zich in jouw aanwezigheid ongemakkelijk of opgelaten kunnen voelen, achter je rug vervelende dingen over je kunnen zeggen, je buiten kunnen sluiten, of je niet vertrouwen.
- ❑ Misschien heb je behoefte aan privacy. Volgens de Autoriteit Persoonsgegevens mag een werkgever niet informeren naar de aard en oorzaak van de ziekte van een werknemer.
- ❑ Een extra argument tegen openheid is volgens mij:

  ____

De belangrijkste argumenten om open te zijn op het werk zijn voor mij:

____

____

____

De belangrijkste argumenten om niet open te zijn op het werk zijn voor mij:

____

____

____

TIP:

Meer informatie over privacy en wet- en regelgeving vind je hier:

- https://www.samensterkzonderstigma.nl/stigma-en-werk/artikelen/bespreekbaarheid-privacy/
- https://www.psynip.nl/actueel/themas/thema/toolbox-werk-en-psychische-klachten/wet-en-regelgeving/wetgeving-toolbox-werk-en-psychische-klachten/

1.2 Wat heb ik nodig?

Nadenken over wat je nodig hebt op het werk of op je werkplek kan een nuttige manier zijn om erachter te komen of je wel of niet wilt vertellen over je psychische gezondheid. Hieronder vind je een lijst met verschillende behoeften die een rol kunnen spelen bij het maken van je keuzes. In de onderstaande lijst kun je de stellingen aanvinken waarmee je het eens bent.

- ❑ Ik heb behoefte aan werkaanpassingen (bijvoorbeeld aangepaste werktijden, andere werktaken, een rustige omgeving).
- ❑ Ik heb ruimte nodig om onder werktijd naar afspraken met mijn arts of behandelaar te gaan.
- ❑ Ik moet verlof op kunnen nemen vanwege mijn psychische gezondheidsproblemen.
- ❑ Ik heb er baat bij als ik (alleen of samen met iemand anders) voor mijn werkgever een plan opstel, waarin staat wat er moet gebeuren als ik ziek word, als ik last krijg van symptomen op mijn werk of als ik verlof moet opnemen.
- ❑ Ik zou graag willen weten tegen wie in mijn werkomgeving ik het beste open kan zijn als ik het wil vertellen.
- ❑ Ik zou meer willen weten over hoe ik erover kan praten; over wat ik wel en beter niet kan zeggen.
- ❑ Ik wil me graag ontwikkelen.
- ❑ Openheid kan helpen bij mijn behoefte om: ____

Ik functioneer het beste op mijn werk of werkplek als:

____

____

____

Ik heb hiervoor het volgende nodig:

____

____

____

1.3 Wat vind ik belangrijk?

Hieronder vind je een lijst met dingen die je belangrijk kunt vinden bij je keuze om wel of niet open te zijn over je psychische gezondheid. Hier kun je de stellingen aanvinken waarmee je het eens bent.

- ❑ Ik vind dat mijn psychische gezondheid een deel van mijn privéleven is. Ik wil dat niet met anderen delen.
- ❑ Ik wil op de werkvloer net als ieder ander behandeld worden.
- ❑ Ik wil mezelf kunnen zijn op m'n werk, zelfs als dit betekent dat mensen me afkeuren of me anders behandelen.
- ❑ Ik vind het belangrijk dat ik op de werkvloer gesteund word.
- ❑ Ik zou graag een voorbeeld willen zijn voor andere mensen met gezondheidsproblemen, zodat zij ook open durven te zijn.
- ❑ Ik vind het belangrijk om door openheid vooroordelen tegen te gaan.
- ❑ Ik zou mijn werkgever en collega's graag willen informeren over wat het betekent om psychische gezondheidsproblemen te hebben.
- ❑ Ik vind het belangrijk om te voorkomen dat mensen medelijden met me krijgen, me betuttelen, me afwijzen of over me roddelen.
- ❑ Ik vind de werkrelatie met mijn werkgever belangrijk.
- ❑ Ik vind het belangrijk om: ____

Voor mij is het in de werkomgeving belangrijk, dat:

____

____

____

TIPS:

Eigen keuzes en het keuzeproces

- Realiseer je dat werkgevers en anderen in de werkomgeving niet alles hoeven te weten. Je weegt zelf af of je je psychische gezondheidsproblemen bespreekbaar wilt maken, en als je dit wilt, wat je tegen wie wilt vertellen.
- Probeer niet in te vullen voor de ander. Je kunt negatieve verwachtingen hebben, terwijl de realiteit meevalt.
- Vaak zijn keuzes over openheid deel van een (herstel)proces. Het zijn meestal geen keuzes die je één keer maakt. Je keuzes kunnen per situatie, persoon en moment verschillen. Het kan helpen om later nog eens terug te kijken naar (delen van) deze keuzehulp.

DEEL 2: Mijn eigen situatie

2.1. Wanneer wel of niet vertellen?

In dit keuzeproces is het nuttig om ook na te denken over het moment waarop je open zou willen zijn over je psychische gezondheid of de gevolgen die deze voor je werk of werkomgeving kan hebben.

Het beste moment om het te vertellen is voor mij (vink jouw keuze aan):

- ❑ Tijdens of rond de sollicitatie.
- ❑ Als ik begonnen ben aan een nieuwe baan.
- ❑ Als ik klachten krijg/heb die van invloed zijn op hoe ik mijn werk doe.
- ❑ Als ik een contract voor onbepaalde tijd heb/in vaste dienst ben.
- ❑ Op een (later) moment, wanneer het relevant is voor de uitvoering van mijn werkzaamheden/functie.
- ❑ Niet.

TIP:
Als je ergens langer werkt, heb je al kunnen laten zien wat je kunt. Veel mensen vinden dat dat een beter moment voor openheid is dan tijdens een sollicitatiegesprek.

2.2 Aan wie wel of niet vertellen?

Sommige mensen vertellen aan niemand over hun psychische gezondheidsproblemen, terwijl anderen bewust uitkiezen aan wie ze het wel of niet vertellen. Weer anderen zijn heel open over hun psychische gezondheid en vinden dat iedereen het mag weten.

Wat op dit moment het beste voor mij zou werken is (vink één strategie aan):

- ❑ **Het geheim houden**: *je vertelt niemand op je werk erover.*
- ❑ **Selectief open zijn**: *je vertelt het een aantal mensen, van wie jij denkt dat ze je zullen steunen of dat ze ervan moeten weten.*
- ❑ **Open zijn zonder onderscheid te maken**: *je maakt je niet druk over wie ervan weet. Je vertelt het aan iedereen die je ontmoet.*
- ❑ **Doelbewust bekend maken:** *je brengt je ervaringen met psychische gezondheidsproblemen over aan een grote groep.*

TIP:
Als je al ergens werkt, kun je misschien inschatten hoe je werkgever of leidinggevende omgaat met psychische gezondheidsproblemen. Hoe open is de cultuur? Is er ruimte om erover te praten? Wat heb je nodig om open te kunnen zijn? Verwacht je dat je werkgever zich flexibel opstelt? Dit kun je meewegen in je afwegingen.

DEEL 3: Tips

In dit deel wordt nog een aantal tips gegeven die je mee kunt wegen in je keuzeproces.

Wat doe, wil en kun je zelf?

- Denk na over wat je er zelf aan kunt doen en al aan hebt gedaan om je werk goed te kunnen uitoefenen.
- Laat zien waar je motivatie ligt om het betreffende werk uit te voeren.
- Geef aan welke werkzaamheden/taken je goed kunt en graag doet en waar je ingezet kunt worden. Een focus op wat je talenten en kwaliteiten zijn en op wat je nodig hebt om die in te kunnen zetten, kan helpen bij het uitvoeren van je werk.
- Vertel wat je nodig hebt om je werk goed te kunnen uitoefenen.

Oefenen

- Als je open wilt zijn, kun je ook eerst oefenen met wat je wilt zeggen, alleen of met iemand die je vertrouwt. Let op de manier waarop je je presenteert (verbaal en non-verbaal gedrag, welke woorden kies je?).
- Door te oefenen met wat je wel en niet vertelt, merk je wat goed werkt voor jou.

Als je het wel vertelt

- Als je het vertelt kan goede voorlichting belangrijk zijn. Je hoeft niet te zeggen wat je 'hebt', maar kunt wel uitleg geven over de eventuele gevolgen op de werkvloer.
- Geef anderen de mogelijkheid te reageren op je verhaal/mededeling

Ondersteuning van anderen

- Bespreek je situatie met vrienden/familie die je in deze volledig vertrouwt.
- Als je niet vertelt over je psychische gezondheidsproblemen, kun je nog wel ondersteuning krijgen van bijvoorbeeld een job coach.
- Probeer jezelf, of wanneer je iemand begeleidt, de ander, niet onder druk te zetten bij het maken van keuzes rond openheid.
- Deze keuzehulp kan de basis zijn voor een goed gesprek over openheid, waarin of waarna je tot je keuzes rond wel of niet vertellen kunt komen.

DEEL 4: Een keuze maken

Je hebt nu de hele keuzehulp doorgenomen en over de volgende dingen nagedacht:

- Wat zijn argumenten (1.1), ondersteuning of behoeften (1.2) en waarden (1.3) die je belangrijk vindt bij keuzes rond bespreekbaarheid in de werkomgeving?
- Je eigen situatie: op welk(e) moment(en) (2.1) en tegen wie (2.2) zou je (eventueel) open willen zijn?
- Tips die je mee kunt nemen bij je keuzes (3).

Bekijk de antwoorden die je aan het eind van deel 1 en 2 hebt gegeven. Deze kun je eventueel bespreken met vrienden, familie, een coach of een behandelaar. Je kunt nadenken over wat je zou willen op de korte en op de langere termijn.

1.a Ik kies ervoor om het niet te vertellen, omdat:

____

____

____

1.b Ik kies ervoor om het wel te vertellen, omdat:

____

____

____

2. Ik kies ervoor om het te vertellen op het moment dat:

____

____

____

3. Ik kies ervoor om het te vertellen aan:

____

____

____

4. Dit is wat ik ga zeggen:

____

____

____

5. Ik wil mijn besluit eerst nog voorleggen aan:

____

____

____

Tot slot

Waar komt de informatie in deze keuzehulp vandaan?

De informatie in de oorspronkelijke Engelstalige versie en de Nederlandse vertaling daarvan - Verzwijgen of Vertellen - is gebaseerd op een uitgebreide literatuurstudie en interviews met mensen die psychische gezondheidsproblemen hebben.

Vervolgens is deze keuzehulp in het project 'Doorontwikkeling CORAL', waarin samengewerkt is door Samen Sterk Zonder Stigma (initiatiefnemer), Kenniscentrum Phrenos en Tranzo, Tilburg University, vernieuwd.

Deze herschreven versie is tot stand gekomen met medewerking van ervaringsdeskundigen/cliënten, professionals op het gebied van Human Resources en arbeidsre-integratie, en werkgevers.

Met wie kan ik contact opnemen over deze keuzehulp?

Als je meer informatie wilt over deze beslishulp kun je contact opnemen met onderzoeker Kim Janssens: k.m.e.janssens@tilburguniversity.edu.
